# *Small Auxin Up RNA*s influence the distribution of indole-3-acetic acid and play a potential role in increasing seed size in *Euryale ferox* Salisb

**DOI:** 10.1186/s12870-020-02504-2

**Published:** 2020-07-03

**Authors:** Zhiheng Huang, Ke Bao, Zonghui Jing, Qian Wang, Huifang Duan, Yaying Zhu, Sen Zhang, Qinan Wu

**Affiliations:** 1 School of Pharmacy, Nanjing University of Chinese Medicine, 138 xianlin Road, Nanjing, 210023 Jiangsu China; 2 Jiangsu Collaborative Innovation Center of Chinese Medicinal Resources Industrialization, Nanjing, 210023 China

**Keywords:** *Euryale ferox* Salisb, Hybrid, RNA-seq, IAA, SAUR

## Abstract

**Background:**

Aquatic *Euryale ferox* Salisb. is an economically important crop in China and India. Unfortunately, low yield limitations seriously hinder market growth. Unveiling the control of seed size is of remarkable importance in improvement of crops. Here, we generated a new hybrid line (HL) with larger seeds by crossing South Gordon Euryale and North Gordon Euryale (WT) which hasn’t been reported before. However, the functional genes and molecular mechanisms controlling the seed size in *Euryale ferox* Salisb. remain unclear. In this study, we focused on the differentially expressed genes in the auxin signal transduction pathway during fruit development between HL and WT to explore candidate regulatory genes participated in regulating seed size.

**Results:**

Both concentration and localization of indole-3-acetic acid (IAA) at two growth stages of fruits of WT and HL were detected by LC-MS and immunofluorescence. Although IAA content between the two lines did not differ, IAA distribution was significantly different. To elucidate the mechanism and to seek the key genes underlying this difference, RNA-seq was performed on young fruits at the two selected growth stages, and differentially expressed genes related to the auxin transduction pathway were selected for further analysis.

**Conclusion:**

Hybrid *Euryale ferox* Salisb. expressed significant heterosis, resulting in non-prickly, thin-coated, large seeds, which accounted for the significantly larger yield of HL than that of WT. Our study indicated that *Small Auxin Up RNA*s (SAURs) -mediated localization of IAA regulates seed size in *Euryale ferox* Salisb. We found that some SAURs may act as a positive mediator of the auxin transduction pathway, thereby contributing to the observed heterosis.

## Background

Euryale semen, also called ‘fox nut’ and ‘Qian shi’ in mandarin, is the seed of *Euryale ferox* Salisb., an important food, ornamental, and medicinal species widely distributed in the southern region of China and North Bihar, India [1]. The species is often considered as an aquatic food due to its high starch content (more than 70%); additionally, in some areas, people prefer Euryale seed to rice for meal because of its low glycemic index [2]. Furthermore, Euryale semen is also a common Chinese traditional medicine whose pharmacological properties including, anti-depression, anti-oxidant, and anti-diabetic action, have been extensively demonstrated by numerous studies [3–5].

**Fig. 1:**
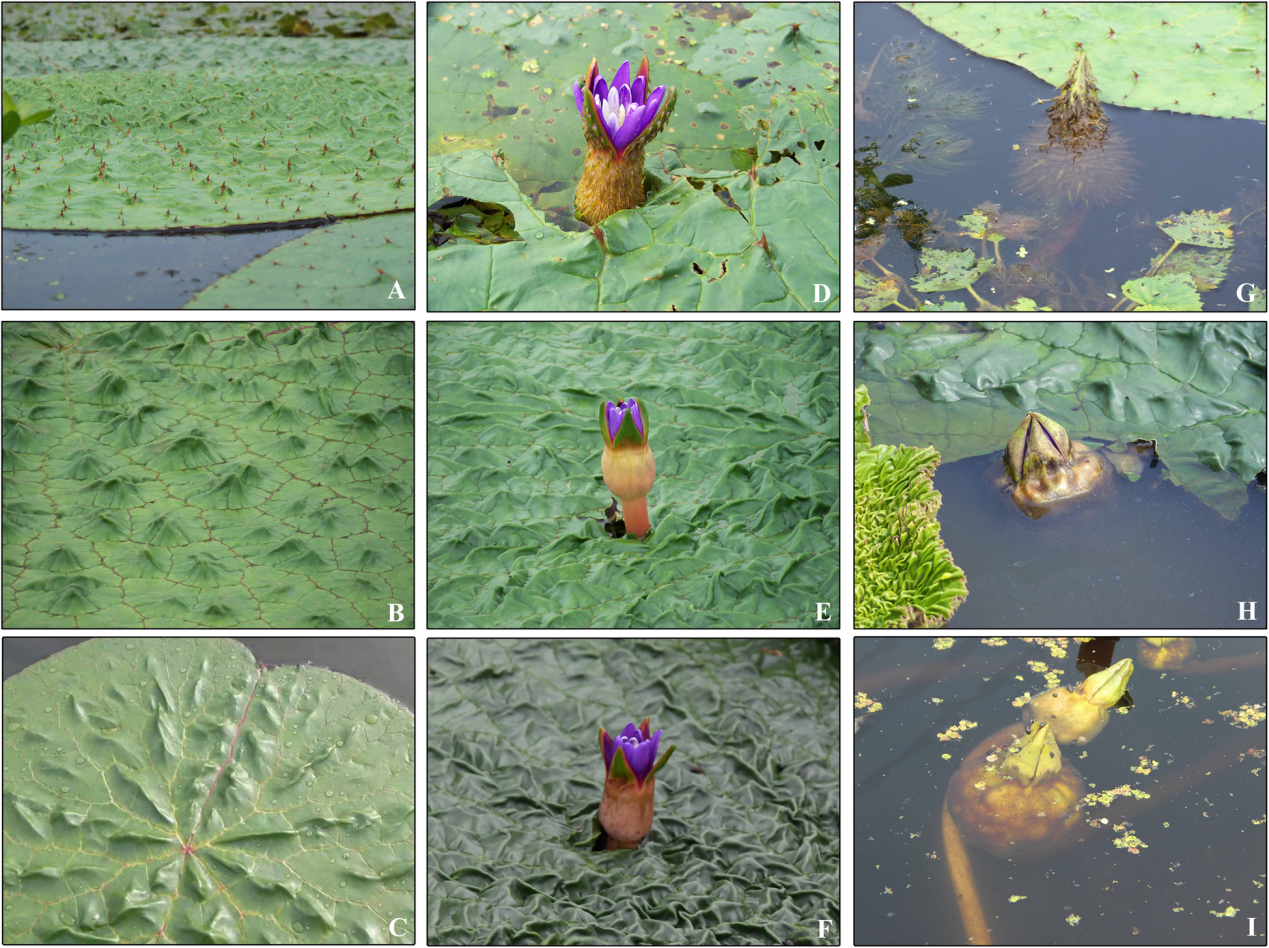
Appearance of WT and SE. The WT has prickly leaves (**a**), flowers (**d**) and fruits (**g**), whereas the SE generates non-prickly leaves (**b**), flowers (**e**) and fruits (**h**). The HL generation has non-pricky leaves (**c**), flowers (**f**) and fruits (**i**) similar to that of SE

*Euryale ferox* Salisb. can be divided into two main types: North Gordon Euryale (wildtype or WT) and South Gordon Euryale (SE) type, as shown in Fig. 1. The WT is distributed in most areas, and bears small, prickly, thin-coated seeds, whereas SE, mainly cultured in Jiangsu and Anhui provinces, bears non-prickly, thick-coated large grains [6]. The yield of the WT is approximately three times higher than that of SE, largely because of the low pod-filling that characterizes SE. However, seeds of SE are tastier than those of the WT, which suggests a starch structural difference between the two types. Therefore, in order to obtain a high yielding crop with tasty seeds, we crossed WT and SE in 2015, and a hybrid line (HL) was successfully developed after self-crossing for three generations. The HL produces thornless tissues and large seeds, which are much more in common with the characteristics of SE, but its growth pattern is more similar to WT (Fig. 1c, f and i). Externally, floating leaves (peltate, > 1.8 m diameter) of HL are green, non-prickly above and red or purple, thorny beneath. Flowers are solitary with four persistent and non-prickly sepals, together with several purple petals. The inferior ovary, below each flower, develops into a spongy berry-like fruit which is non-prickly, and each mature fruit contains 60–90 seeds with thin black seed coats. HL is advantageous for having a higher yield, with the average yield of HL in experimental plots up to 4875 kg ha^− 1^, 35% greater than the yield of the WT in 2018. However, the seed number per plant did not show any difference between the WT and HL, indicating the yield evaluation in HL was mainly attributed to the size and weight of seeds. In a previous study, we did not detect any difference between WT and SE at the DNA level, which suggested both types belong to the same species, whereby transcriptional differences must be the cause for the phenotypic differences between them.

Endogenous phytohormones are well known to play critical roles in plant physiological and biochemical processes at the molecular level [7–9]. Among plant growth regulators, auxin (indole-3-acetic acid, IAA) regulates whole plant developmental processes [10, 11]. IAA affects cell division and cell elongation; furthermore, some yield traits in all higher plants are largely dependent on this small organic-acid molecule [12]. Plant architecture has been demonstrated to be directly related to yield and to be controlled by pin-formed5b (PIN5b) through changes in IAA homeostasis, transport, and distribution [13]. Further, these roles of IAA have been demonstrated in many genetic studies on the regulation of IAA through control of the metabolism of IAA, changes of IAA distribution pattern and on plant responses to IAA signaling [14, 15]. Furthermore, it is increasingly interesting to investigate the role of genes included in IAA-signal transduction pathway in biological process mediated by auxin [16]. Up to present, a large number of IAA-related factors included in IAA-signal transduction pathway have been documented in plants, including the F-box TRANSPORT INHIBITOR RESPONSE 1/AUXIN SIGNALING F-BOX PROTEIN (TIR1/AFB) auxin co-receptors, the AUXIN RESPONSE FACTOR (ARF) transcription factors, and the Auxin/INDOLE-3-ACETIC ACID (Aux/IAA) transcriptional repressors [17, 18]. Auxin stimulates the interaction between TIR1/AFB and Aux/IAA proteins and then induces the degradation of Aux/IAA and the release of ARF factors, thereby contributing to the rapid transcription of a set of auxin-responsive genes which implicate in diverse processes in plants, such as embryogenesis and organogenesis [19, 20]. Thus, the *Small Auxin Up RNA* (*SAUR*), *Glycoside Hydrolase 3* (*GH3*) and *AUX/IAA* genes are three primary auxin-responsive gene families; unfortunately, functional studies on SAURs have lagged behind [21]. The reported genetic functions of SAURs include elongating soybean hypocotyl sections and promoting Arabidopsis hypocotyl and stamen filament elongation, which indicate a role for SAURs in the regulation of cell expansion [22–24]. In a previous study, OsSAUR39 was shown to influence rice yield through mediating auxin synthesis and transport, implying a potential ability of SAURs to regulate crop yield [25–27]. It’s not clear whether the SAURs and IAA-signal transduction pathway is related with the increased seed size in HL.

Due to the distribution of WT is much broader than the distribution of SE in China and India, and most studies investigated formerly used WT as experimental material, only WT was thereby employed as a wild-type control in this study [28, 29]. To explore the potential functional genes for the promotion of seed size in *Euryale ferox* Salisb., we subjectively divided growth into stages I through V according to fruit growth regulation in *Euryale ferox* Salisb., after observing largely difference in phenotype and IAA distribution between WT and HL. Then, RNA-seq was performed on fruits at stages II and III to identify the differentially expressed genes (DEGs) involved in the IAA signal transduction pathway. These results indicated that SAURs-mediated localization of IAA regulates seed size in *Euryale ferox* Salisb.

## Results

### HL produces larger seeds and kernels

**Fig. 2:**
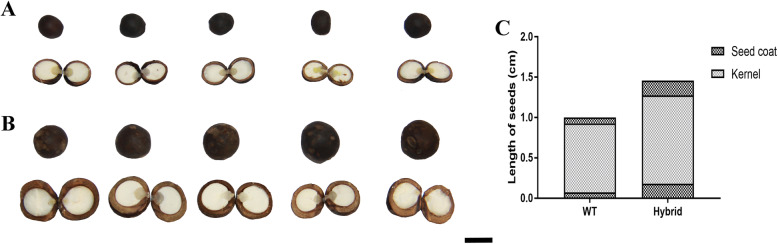
The seed size of WT and HL was measured. The obviously differences between WT (**a**) and HL (**b**) were observed in size of seeds, kernels and coats (bar = 1 cm). And (**c**) length of both kernel and coat in seed of HL are significantly larger than in WT

After growing for 21 weeks, the two plant types matured and their seeds were collected. It is worth noting that the seed number per plant did not show any difference between the two lines in our data (on average, WT was about 1170 seeds per plant, while HL was 1180), which indicated that the yield difference between these two lines was probably attributed to seed size and weight. Although the weight per seed of HL (range from 1.1 g to 1.7 g) was much heavier than that of WT (range from 0.5 g to 0.9 g), we paid more attention to seed size than to weight because, during the growth of reproductive, weight gain is most noticeable in the rapid changes in seed size [30]. All seeds were subjected to a brief drying period in the sun, and then seed size and kernel number of WT and HL were recorded. As shown in Fig. 2, HL (B) produced larger kernels than WT (A). As Fig. 2c shows, although the seed coat of HL was obviously thicker, the large size of the seeds contributed to larger kernels in HL.

### The difference in fruit length between the two experimental lines became significant at growth stage III

**Fig. 3:**
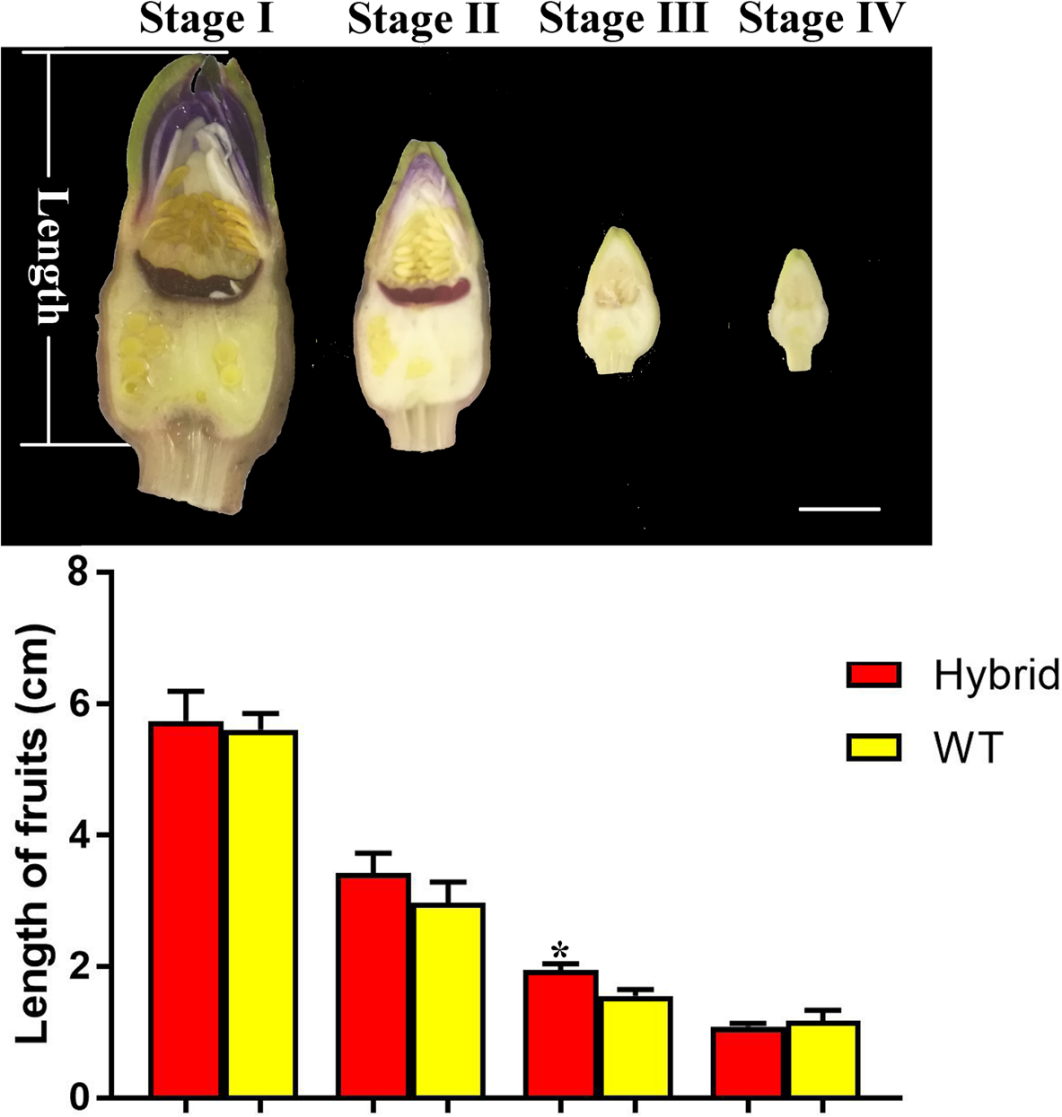
On the 10th week, the length of fruits at different stages was measured. Fruits in HL (top of the Fig. 3) grew significantly faster than in WT at stage III among four stages. * *P* < 0.05, compared to WT (bar = 1 cm)

To further understand the growth regulation of *Euryale ferox* Salisb., we divided development into five stages (I through V), as in rice studies [31, 32]. At stage I, fruits grow at a slow pace; at stage II, no significant growth in fruit size has occurred; at stage III, fruits show fast growth; at stage IV, fruits are nearly mature, and, at stage V, fruits have entered post-maturity (Fig. S1). We collected all fruits on the 10th week according to the growth pattern, and, more importantly, fruit growth at this phase contributed to the differences between the two lines later in development. As shown in Fig. 3, fruit length in HL was greater than in WT at stages III (*p* < 0.05), consequently, we selected samples at stages II and III (fruit width can be found in Fig. S2). Further, both HL and WT produced 1–2 fruits above water level per week.

### Localization of IAA in HL differed from that in WT

**Fig. 4:**
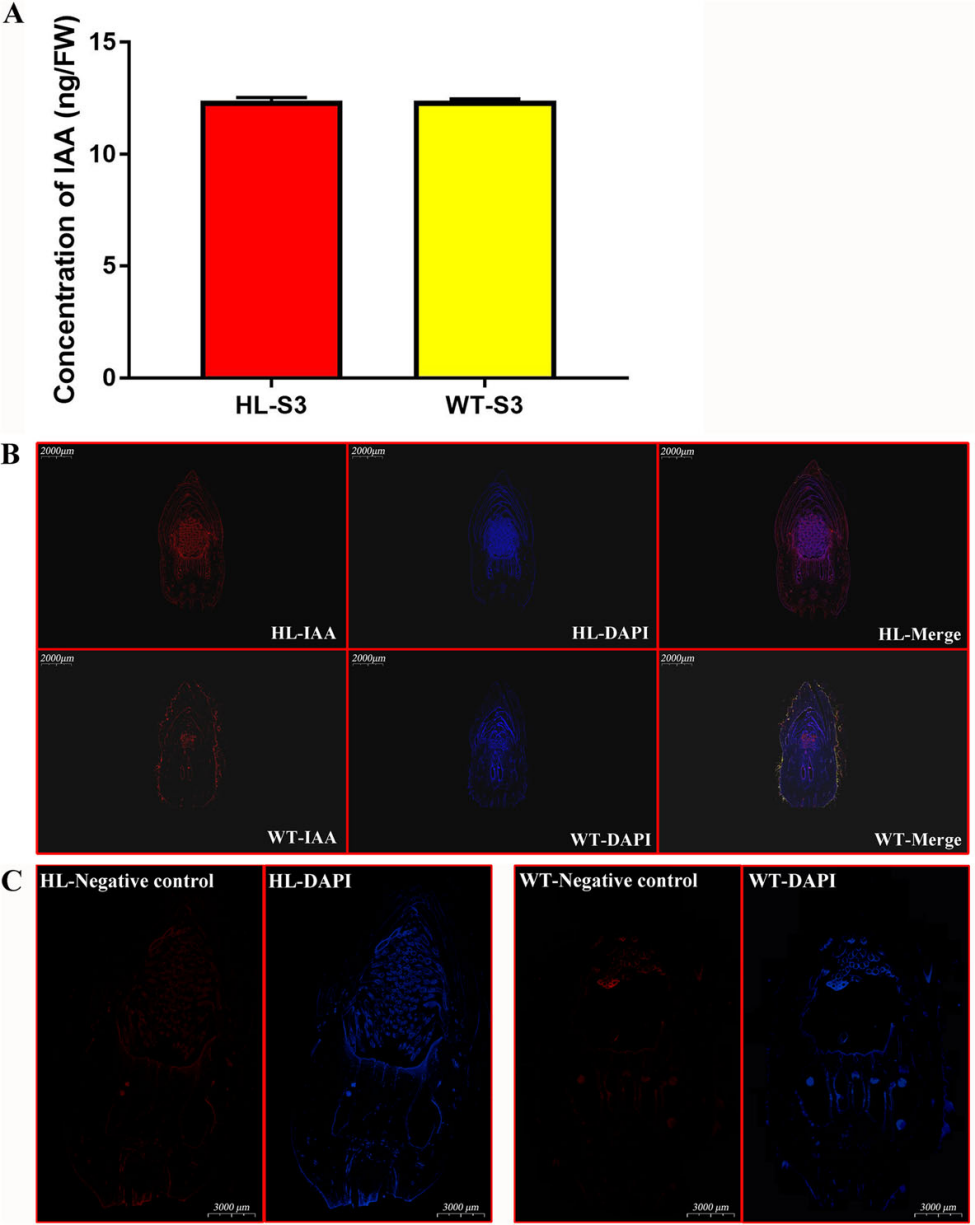
Concentration and distribution of IAA in fruits at stage III were detected by LC-MS and immunofluorescence. (**a**) Fruits in HL and WT did not show significant difference in concentration of IAA at stage II, while (**b**) the localization of IAA (red) in WT was partly distributed in prick. S3, stage III. (**c**) Negative control

Phytohormones play important roles in both plant growth and crop yield. IAA is one of the most active effectors in young tissues [33]. We measured IAA content at the initial growth stages and found no difference between fruits of the two lines at stage III (Fig. 4a). However, both concentration and localization of IAA at the cellular level, which must control cell activity and/or its fate, are critical [34]. Therefore, to investigate the localization of IAA in fruits at the same stage, immunofluorescence staining was performed on sectioned fruits. Surprisingly, at stage III, WT allocated free-IAA to its special tissue, pricks, more frequently, whereas the fluorescence signal in fruits of HL concentrated in the ovary and the stamen (Fig. 4b). Similar results were found at stage II (Fig. S3), and the negative control could be found in Fig. 4c. This most interesting finding suggested a potential role for auxin signal response in regulating ovary size and, subsequently, seed size. We also detected the localization of free-IAA signal in SE fruits with similar size (Fig. S4). It was found that free-IAA only located in stamens of SE fruit, indicating different utilizations of IAA among three types. However, it’s also possible that SE fruit (even if with similar size) was at later growth stage, because the fruit of SE grows much faster than that of WT and HL.

### RNA-seq and transcriptome assembly

A series of de novo assemblies were carried out with Trinity. To obtain useful transcriptomic information for the two lines during development, 12 cDNA libraries were established and paired-end sequence reads were generated using the Illumina Hiseq X10. As shown in Table 1, a total of 630,221,090 clean reads were generated from HL and WT cDNA libraries. The average base fraction with quality scores of Q30 and Q20 were 94.6 and 98.1%, which indicated high quality data. Relative length of assembled sequences is an important evaluation criterion for assembly quality, and the summary statistics of the assembled clean reads are shown in Table 2. Using the Trinity assembler, 197,253 unigenes were generated with an average length of 959 nt, an N50 of 1574 nt, and a total length of 189,170,382 nt. Total unigenes included 44,511 (22.57%) of less than 300 nt, 151,012 (76.55%) with lengths from 301 to 5000 nt, and 1730 (0.88%) with lengths greater than 5000 nt These results proved high quality sequencing and assembly in our samples.

**Table 1:** Statistical Analysis of Transcriptome Sequencing Data

Sample	Clean reads number	Clean bases	Clean rate(%)	Q20(%)	Q30(%)
WT-S2–1	49,343,466	7,392,663,824	95.64	97.78	93.69
WT-S2–2	35,660,154	5,349,023,100	95.3	98.03	94.77
WT-S2–3	46,271,622	6,946,934,906	94.97	97.84	93.84
WT-S3–1	38,688,882	5,803,332,300	95.66	98.11	94.98
WT-S3–2	53,603,992	8,023,343,356	97.29	97.69	93.01
WT-S3–3	65,400,866	9,791,552,137	96.21	97.77	93.57
HL-S2–1	106,884,270	16,025,803,643	97.05	97.96	93.96
HL-S2–2	31,283,988	4,692,598,200	95.41	98.07	94.89
HL-S2–3	48,644,712	7,296,706,800	96.35	98.42	95.62
HL-S3–1	42,801,744	6,420,261,600	96.21	98.4	95.59
HL-S3–2	40,785,442	6,117,816,300	96.05	98.43	95.66
HL-S3–3	46,852,120	7,027,818,000	96.41	98.43	95.66

**Table 2:** Length distribution of the unigenes in transcriptome

Length range	Unigenes	Percentage (%)
200–300	44,511	22.57
300–500	43,507	22.06
500–1000	48,729	24.70
1000-2000	36,800	18.66
2000-5000	21,976	11.14
5000+	1730	0.88
Total number	197,253	
Total length	189,170,382	
N40 length	1980	
N50 length	1574	
N60 length	1220	
N70 length	910	
N80 length	633	
N90 length	390	
Min length	201	
Max length	17,279	
Mean length	959	

### Annotation and GO classification

**Fig. 5:**
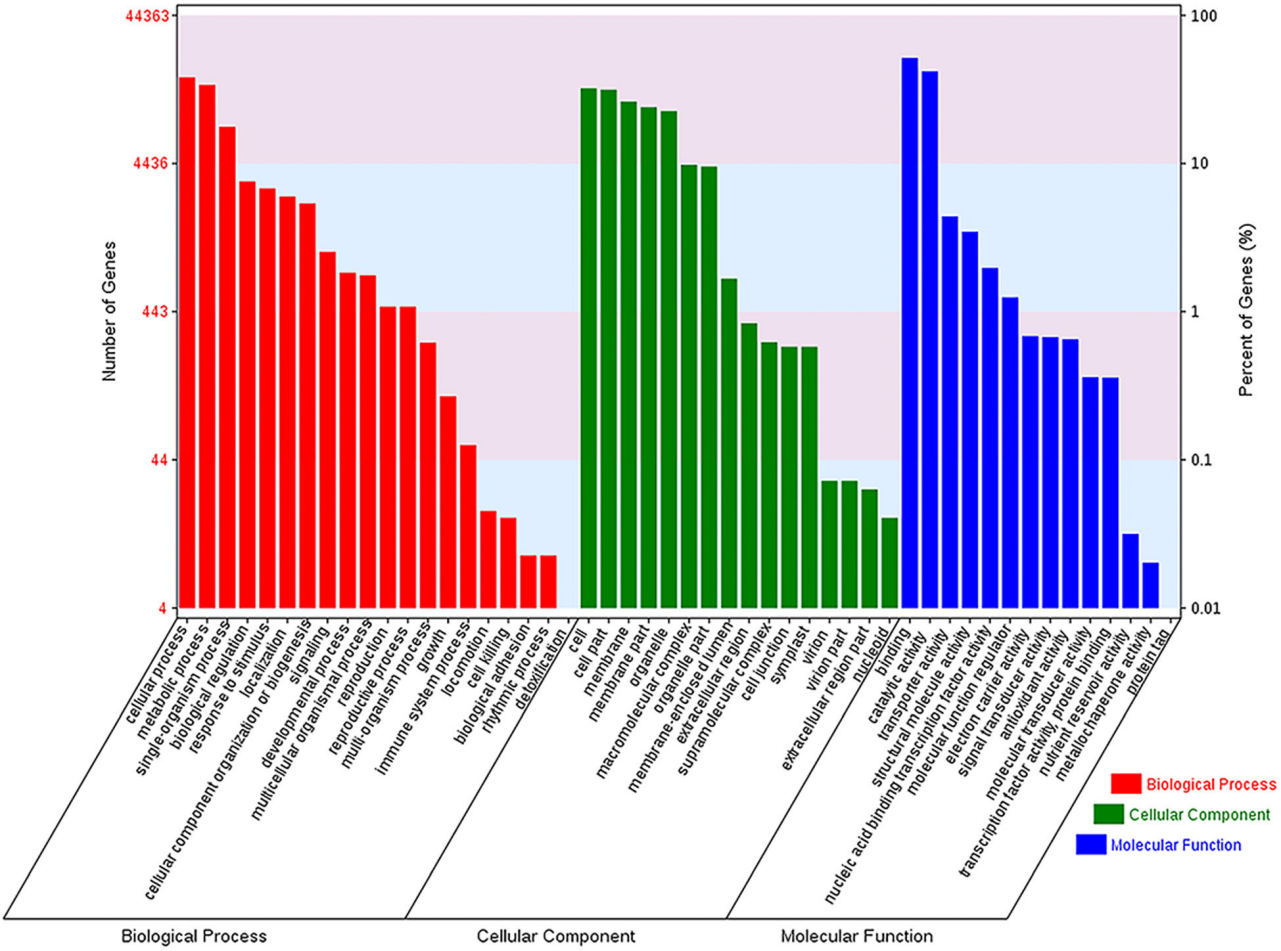
Functional annotation of assembled sequences based on GO categorization. 44,363 unigenes were grouped into “cellular component”, “molecular function”, and “biological process”

The annotation of the 197,253 assembled unigenes revealed that 44,363 (22.49%), 18,229 (9.24%), 26,746 (13.56%), 9633 (4.88%), 3632 (1.84%), and 2879 (1.46%) showed significant similarity in the GO, Pathway Pfam, TMhmm, Eggnog, SignalP, and Uniprot databases, respectively. In all, 71,399 unigenes (36.20% of all unigenes) were successfully annotated in at least one database (Table 3). As shown in Fig. 5, total 44,363 unigenes annotated in GO were assigned to three principal domains; “cellular component”, “molecular function”, and “biological process”.

**Table 3:** Unigenes annotated in databases

Database	Number of unigenes	Percentage (%)
GO	44,363	22.49
Pathway Pfam	18,229	9.24
TMhmm	26,746	13.56
Eggnog	9633	4.88
signalP	3632	1.84
Uniprot	2879	1.46
All database	68,200	34.57
At least one database	71,399	36.2
Total unigenes	197,253	100

### SAURs induced differences in the IAA signal transduction pathway between HL and WT

**Fig. 6:**
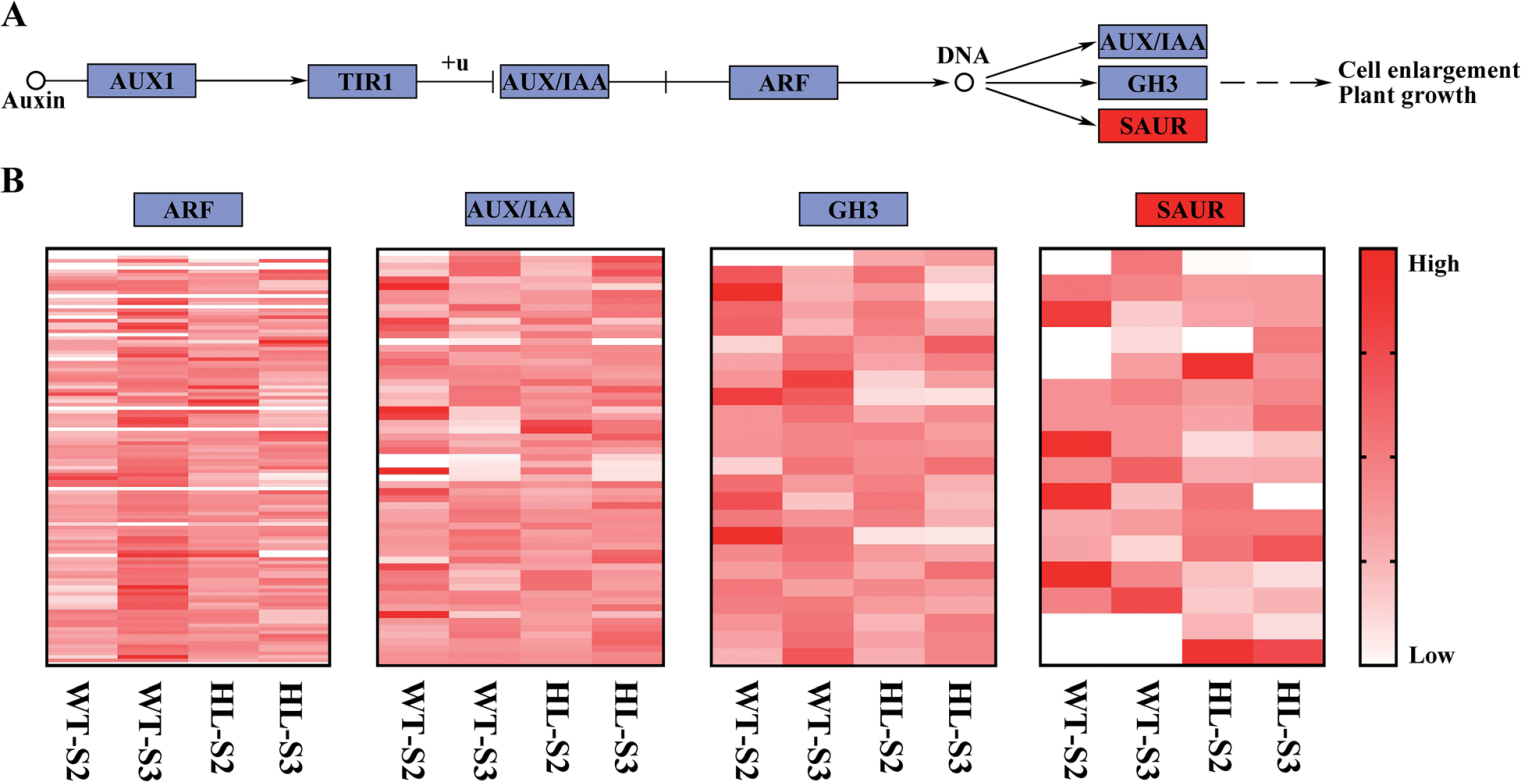
DEGs in the IAA signal transduction pathway between HL and WT at stage II and III. (**a**) DEGs of HL and WT involved in auxin transduction pathway were marked with red, and (**b**) the relative expression of ARFs, AUX/IAAs, GH3s, SAURs were analyzed. S2, stage II; S3, stage III

The KEGG Pathway database records the networks of molecular interactions in cells and species-specific variations [35]. According to the findings on localization of IAA reported above, we focused on genes involved in the auxin transduction pathway. The pathway is shown in Fig. 6a; briefly, auxin promotes an interaction between TIR1/AFB and Aux/IAA proteins, resulting in the degradation of the Aux/IAAs and the release of ARF repression, and down-stream SAURs, GH3 and AUX/IAA are activated subsequently. We compared the transcriptome from HL with that from WT at both stages II and III: only SAURs showed a significant difference in the whole pathway (Fig. 6a). Then, all unigenes annotated with *ARF*, *AUX/IAA*, *GH3* or *SAUR* were selected for comparison (Fig. 6b). An obvious difference was observed in SAURs, while the other three families did not show any changes between the two lines at either growth stage. It is worth noting that the unigene number of SAURs is the least among the four families, which indicated less functional redundancy in SAURs of *Euryale ferox* Salisb. Interestingly, this finding suggests an important role for SAURs in responding to IAA and, probably, up-regulating seed size in HL.

### Verification of RNA-seq by RT-qPCR

**Fig. 7:**
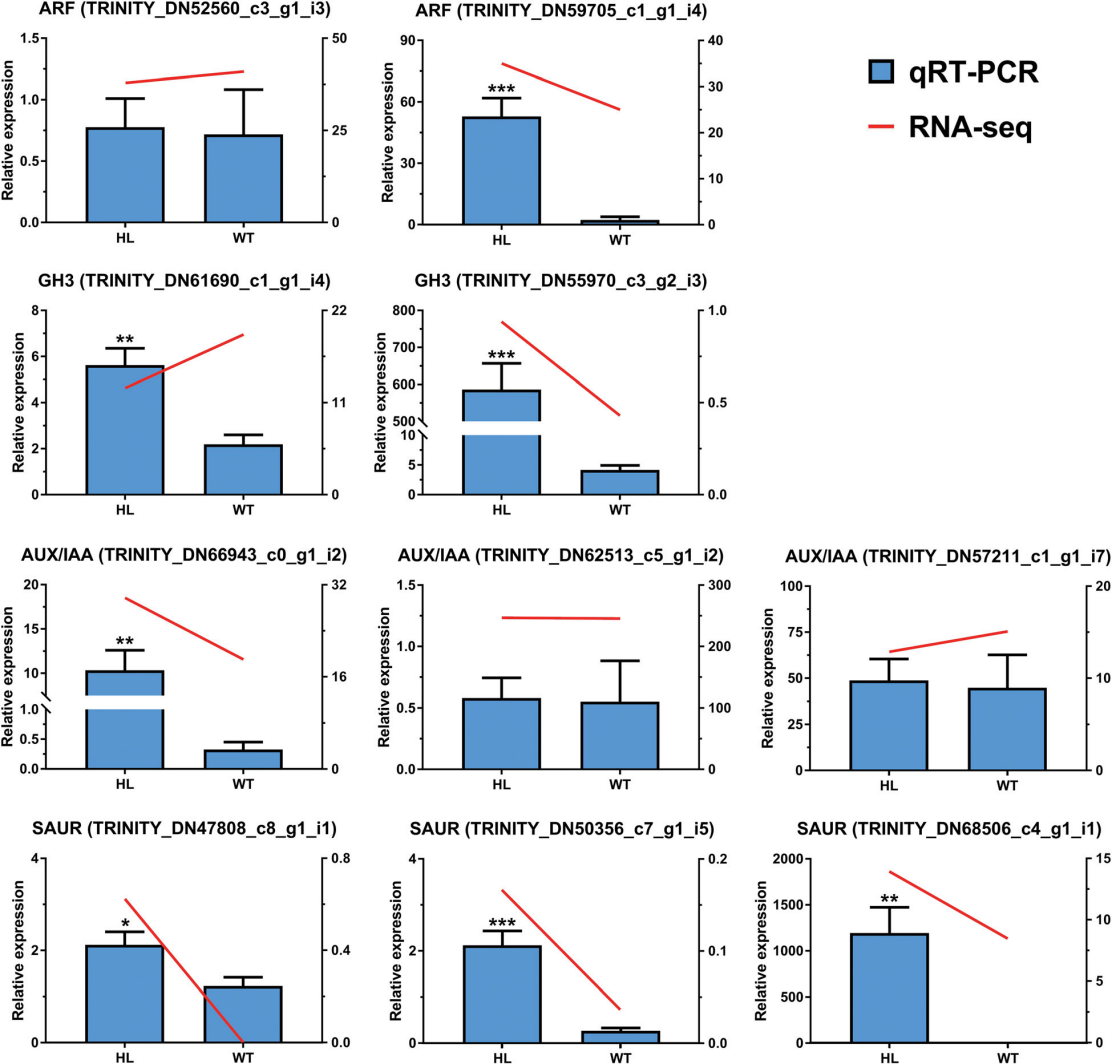
Relative expression levels of 10 candidates were detected by RT-qPCR to verify the results from RNA-seq. Most candidates in RT-qPCR showed similar expression pattern compared to RNA-seq. *n* = 4–6, * *P* < 0.05, ** *P* < 0.01, *** *P* < 0.001, compared to WT

To evaluate the validity of the expression of DEGs, 10 candidate unigenes involved in the auxin transduction pathway (2-ARFs, 3-AUX/IAAs, 2-GH3s, and 4-SAURs) were detected by RT-qPCR (Detailed information about all 10 unigenes is given in Table S1.). Fruits from HL and WT at stage III were selected for RT-qPCR analysis, and β-actin was used as a reference gene in this study. As shown in Fig. 7, most of these DEGs showed a similar expression pattern according to RT-qPCR and RNA-seq results, which suggested that results from the transcriptome data are reliable.

## Discussion

Hybrids often present phenotypes that surpass their parents in terms of growth and yield, a phenomenon known as “hybrid vigor” or “heterosis” [36]. Hybrid rice largely contributes to solve the problem of food for hundreds of millions of people all over the world due to heterosis effects on yield [37]. Recently, with the improvement of living standards, people pay more attention to health and tasty food. *Euryale ferox* Salisb. is considered as one suitable substitute for rice and wheat as it is widely distributed and has a waxy taste [38]. However, development of this crop faces many problems, such as high planting costs and low yield. To overcome these problems, we spent many years selecting dominant lines and crossbreeding them, until finally, a HL was developed. To our surprise, compared to the WT, the new hybrid exhibited significantly higher yield; in addition to which, it showed a number of advantageous traits, including non-prickly and heavily waxy seeds. After comparing this new HL with parent WT for yield traits, the size of kernel generated by the HL was much larger than that of WT, which revealed kernel size as a crucial factor in improving the yield of HL. Besides, the high yield of HL was also attributed to its heavier seeds, but seed weight primarily depends on seed size especially during the process before seed filling, indicating a crucial role for seed size in evaluation of yield of *Euryale ferox* Salisb. during the growth of its reproductive organs [39, 40]. Seeds of aquatic plants often have higher moisture water, which induces a large error in seed weight [41]. As a consequence, seed size was taken as the main observation in this study to investigate functional genes and molecular mechanisms in *Euryale ferox* Salisb.

To establish a growth pattern of *Euryale ferox* Salisb., we divided the phases of fruit development into five stages according to average fruit growth above the water. Fruit growth rate is low over the first two stages, especially at stage II, which suggests that during these stages, vegetative and reproductive organs grow together. In contrast, over the remaining three stages, reproductive growth allows only fruit generation and growth [42]. The significant decrease in growth at stage V may be due to water loss in fruits during post-maturation [43]. The growth stage division of HL was based on rice studies and was critical for sample selection and time course studies of HL and WT. As expected, we observed a significant difference in fruit growth rate between the two lines under study. Factors that contributed to this difference became the focus of our study.

Although heterosis has been successfully applied to increase HL yield, the molecular mechanisms involved remained unknown [44]. As the difference in growth induced by heterosis can be largely revealed by transcriptome analysis, we performed RNA-seq analysis on the two lines [45, 46]. Concentrations of auxin varied across different tissues, mediating distinct developmental outcomes and contributing to the functional diversity of auxin [47]. In our study, IAA content at the early stage did not show any difference between the two plant materials, whereas IAA localization at the same selected growth stages was different for the two lines. Plant tissues rapidly sense and respond to changes in auxin levels. These responses involve several major classes of auxin-responsive genes [48]. We hypothesized that, in WT, at this early stage pricks happen to constitute a protective natural barrier, and this process uses considerable amounts of IAA, whereas HL invests IAA only on developing ovaries and stamens. This also explains why the ovary of HL is somewhat larger than that of WT. We speculate that the elongation effect of IAA on the ovary in HL was influenced by the difference in IAA allocation pattern between HL and WT. In SE fruit, IAA signal mainly distributed near stamens, this is probably because SE grows faster than WT and HL, and with a longer period of flowering days. Furthermore, it is worth noting that this process is probably mediated by SAURs as, in the auxin transduction pathway, only this gene family was detected as a differentially expressed set of genes between the two lines at both growth stages. This hypothesis is supported by the fact that some SAURs promote cell expansion in plant tissues [49–51]. And many genes in IAA signal transduction pathway have been reported to affect the seed development [52, 53]. However, there were also a few genes involved in *GH3* that were found upregulated in HL due to the large number of unigenes in GH3. These differences did not induce a significantly different expression of GH3s. Previously, the transcriptomic analysis in seed samples of *Euryale ferox* Salisb. suggested that PAL and P450-related genes promote the maturation of seed by affecting the phenylpropanoid biosynthesis, indicating a crucial role for genes or pathways in the promotion of development of *Euryale ferox* Salisb [54]. Differ from previous study, we used fruits of both WT and HL types for RNA-seq analysis and considered IAA-related genes as the potential function genes in seed development. Our study not only provided more complete reads and more precise annotations of unigenes in *Euryale ferox* Salisb. but also indicated a potential mechanism in promoting seed development.

Summary, though phenotypic observation, IAA detection and RNA-seq analysis, our results gradually revealed that SAURs potentially mediated the localization of IAA and thereby regulated seed size in HL. In addition to which, we also provided a preliminary investigation on functional genes and molecular mechanisms involved in seed formation of *Euryale ferox* Salisb.

## Conclusions

Hybrid *Euryale ferox* Salisb. expressed significant heterosis, resulting in non-prickly, thin-coated, large seeds, which accounted for the significantly larger yield of HL than that of WT. Through the study, we found that some SAURs may act as a positive mediator of the auxin transduction pathway, thereby contributing to the observed larger seed. The gene functions of these SAURs in *Euryale ferox* Salisb., and the underlying mechanisms deserve further investigation.

## Methods

### Plant materials and growth conditions

*Euryale ferox* HL and its parents, North Gordon Euryale (*E. ferox*, WT) and South Gordon Euryale (*E. ferox*, SE), were used as plant materials in this study. Seeds of WT were originally obtained in 2001 from the wild (Gaoyou lake of Jiangsu province) and cultured in the experimental field of Jiangsu Seed and Seed Breeding Base, and seeds of SE were originally obtained from the wild (Suzhou of Jiangsu province) and also grown in the experimental field of Jiangsu Seed and Seed Breeding Base who provided permission for their use in this scientific research. HL (containing 3 lines) were constructed from WT as the female crossed with SE to generate F_1_ hybrid in Gaoyou (E119°30′, N32°58′), Jiangsu province, China in 2015; later, F_1_ was selected and self-pollinated to generate F_2_ in Nanzha (E119°10′, N33°18′), Jiangsu province, China in 2016; and F_2_ individuals were used to produce F_3_ by self-fertilization in Gaoyou (E119°30′, N32°58′), Jiangsu province, China in 2017; finally, F_3_ generation and both of its parents were cultured in the experimental field of Jiangsu Seed and Seed Breeding Base (Gaoyou, Jiangsu province). All plant materials used in this study were supplied by the Jiangsu Seed and Seed Breeding Base. Collection of all samples completely complies with national and local legislation permission, and no specific permission was required for collecting these plants. The plant materials were formal identified by Professor Qinan Wu, and a voucher specimen of this material has been deposited in Nanjing University of Chines Medicine. HL and the WT were sown in a completely randomized block design with three replications in April 22, 2018. Each plot consisted of 160 individual lines, each separated 2 m from its neighboring lines. The two lines were selected in this study to measure phenotypic traits and conduct transcriptome analyses. The young fruits selected for sampling at the designated development stages were collected and stored at − 80 °C for RNA-seq and RT-qPCR analysis. Seeds were collected at maturity on September 9, 2018; they were selected with 10 replicates for yield traits; additionally, each sample had at least three biological replications to minimize systematic errors.

### IAA quantification

IAA was quantified by liquid chromatography-mass spectrometry (LC-MS), essentially as described [55]. Briefly, 50 mg of frozen tissue was homogenized in 500 μL of extraction buffer (isopropanol: double distilled water: acetic acid = 2:1:0.002, vol/vol/vol). After shaking on ice for 30 min, 1 mL dichloromethane was added to each sample and shaken for another 30 min. Then, samples were centrifuged at 13,000 g for 5 min at 4 °C; the lower phase was dried under a stream of nitrogen gas, and redissolved with methyl alcohol. Each sample solution was injected into the reverse-phase C_18_ HPLC column (2.1 mm × 100 mm, 2.7 μm, Agilent, USA) for LC-MS (Shimadzu LC-30 ultrahigh performance liquid chromatography system, Shimadzu, Japan; AB Sciex QTRAP 5500, SCIEX, USA) analysis. An IAA standard (≥98%, 45,533, Sigma, USA) was used as the external standard.

### Immunofluorescence staining

Immunofluorescence staining for IAA was performed on sections of fruits as follows: Briefly, 60 μm fruit sections were cut using a cryotome (CM1950, Leica Microsystems, Germany) and affixed onto glass slides, followed by dehydration in ascending and descending ethanol solutions. After washing three times for 10 min in phosphate buffer saline (PBS) buffer with 0.1% (v/v) Tween 20, sections were pre-treated with 5% (w/v) bovine serum albumin in PBS for 1 h at room temperature to reduce non-specific binding, followed by incubating overnight with anti-IAA antibody (1:200, AS09 421, Agrisera, Sweden) at 4 °C. After washing three times with 0.1% (v/v) Tween 20 in PBS buffer for 10 min, sections were incubated with Alexa Fluor 594 (1:200, conjugated Goat Anti-Rabbit IgG (H + L), SA00006–4, Proteintech Group, USA) as a secondary antibody for 1 h in darkness at room temperature. Samples were washed three times for 10 min with 0.1% (v/v) Tween 20 in PBS. Sections were imaged using a Carl Zeiss Axio Scope fluorescence microscope (Gottingen, Germany). Samples incubated with PBS instead of primary antibodies were used as negative controls, and there was no unspecific binding of the secondary antibody in this study.

### RNA extraction, cDNA library preparation and sequencing

Before total RNA was extracted from samples, 12 frozen tissue samples were fully grinded separately under liquid nitrogen. RNA quality was determined by Agilent 2100 BisAnalyzer (J06–02, Agilent, USA), and samples with both RIN ≥ 7 and $\frac{28S}{18\mathrm{s}}\ge 1.5$ were included for RNA-seq analysis. RNA concentration was determined using a Qubit RNA BR Assay Kit (Q10211, Invitrogen, USA).

To construct the cDNA libraries, 5 μg total RNA per sample was used as the template. The NEBNext Ultra RNA Library Prep Kit for Illumina (E7530S, NEB, USA) was used to generate a series of corresponding libraries, and index codes were applied to each sample. Briefly, mRNA was purified from total RNA using oligod (T) beads. RNA fragmentation was carried out at high temperature in the presence of divalent cations in NEBNext First-Strand Synthesis Reaction Buffer (5X). First-strand cDNA was synthesized using random hexamer primers and M-MLV Reverse Transcriptase. Second-strand cDNA synthesis was subsequently performed using DNA polymerase I, RNase H, dNTP and buffer. Then, the cDNA fragments were purified, end repaired, and poly (A) added, followed by adapter ligation. The selected ligation product was amplified and, subsequently, sequenced with Illumina HiSeq TM X10 by Gene Denovo Biotechnology Co. (Beijing, China).

### Identification of differentially expressed mRNAs

After high-throughput sequencing, raw reads with too many unknown bases (> 5%) or low-quality bases (> 30% of the bases with a quality score ≤ 20) were excluded to obtain clean reads. The remaining clean reads from each sample were then assembled by Trinity^3^ with de Bruijn graph.

The unigene sequences were annotated using the following public databases: Universal Protein (Uniprot), evolutionary genealogy of genes (Eggnog), Pfam protein domain database (Pfam), signal peptide server (SignalP), Hidden Markov Model-based transmembrane protein database (TMhmm), Kyoto Encyclopedia of Genes and Genomes (KEGG), and Gene Ontology (GO). The unigene sequences were aligned using BLASTx with an e-value of < 10^− 5^, and the annotations were only saved when the “-max_target_seq” was “1”.

Differential expression was estimated and tested using the RSEM^12^ software with the EM method. We quantified gene expression levels in terms of Fragments Per Kilo bases per Million (FPKM), calculated the false discovery rate (FDR), and estimated the fold change (FC) and log_2_ values of FC. Transcripts that exhibited an FDR ≤ 0.01 and an estimated absolute │log_2_(FC) │ ≥ 1 were considered to be significantly differentially expressed.

### Quantitative real-time PCR (RT-qPCR) analysis

RNA was extracted using TRIzol (T9424, Sigma, USA) and 1 μg used for cDNA synthesis with the PrimeScript RT Master Mix kit (RR036A, TaKaRa, China). RT-qPCR was performed with a QuanStudio 3 instrument (Applied Biosystems, USA), using 5 μL cDNA, NovoStart SYBR qPCR SuperMix Plus (E096-01B, Novoprotein, China), and 0.2 μL each of two gene-specific primers (Table S1) in a final volume of 20 μL. The thermocycling regime consisted of 2 min at 50 °C, 10 min at 95 °C, followed by 40 cycles of 15 s at 95 °C, 1 min at 55 °C, and 30 s at 72 °C. Disassociation curves verified amplification of a single product. The relative expression of each gene was analyzed using the comparative Ct (ΔΔCt) method with β-actin as a reference gene for normalization. The reference gene was selected according to previous studies [56]. Briefly, gene-specific primers were designed by Primer Premier 5 and checked by melting curve after amplification. The expression stability of each candidate reference gene was compared and ranked by BestKeeper software according to the threshold cycle (Ct) values. Reference genes with a standard deviation value below 1 were considered as stably expressed in this study, and a smaller coefficient of variation indicates a more stable reference gene. Each amplification reaction was performed in triplicate.

### Data analysis

All data were expressed as the mean ± standard error of the mean. Differences between two groups were analyzed using *t-test* and *P* < 0.05 was considered to indicate a statistically significant difference. Statistical analysis was performed with GraphPad Prism 6.0 software (GraphPad Software, Inc.).

## Supplementary information

**Additional file 1: Figure S1.** The development stage division of fruits.

**Additional file 2: Figure S2.** Width of fruits in HL and WT.

**Additional file 3: Figure S3.** Concentration and distribution of IAA at stage II.

**Additional file 4: Figure S4.** Distribution of IAA in fruits of SE.

**Additional file 5: Table S1.** Detail information of Unigenes.

## Data Availability

The datasets used and/or analyzed in the current study are available from the corresponding author upon reasonable request. The role of the funding body in the design of the study and collection, analysis, and interpretation of data and in writing the article should be declared in this request. All Illumina Sequencing data have been deposited in NCBI Sequence Read Archive (SRA) under accession number: PRJNA628746 (https://www.ncbi.nlm.nih.gov/bioproject/PRJNA628746).

## Abbreviations

HL: Hybrid line
WT: North Gordon Euryale (wild type)
IAA: Indole-3-acetic acid
TIR1/AFB: Transport Inhibitor Response1/Auxin Signaling F-box Protein
ARF: Auxin Response Factor
*SAUR*: *Small auxin up RNA*
*GH3*: *Glycoside Hydrolase 3*
*AUX/IAA*: *Auxin/Indole-3-Acetic Acid*
DEG: Differentially expressed genes
LC-MS: Liquid chromatography-mass spectrometry
PBS: Phosphate buffer saline
Uniprot: Universal Protein database
Eggnog: Evolutionary genealogy of genes database
Pfam: Pfam protein domain database
SignalP: Signal peptide server database
TMhmm: Hidden Markov Model-based transmembrane protein database
KEGG: Kyoto Encyclopedia of Genes and Genomes database
GO: Gene Ontology database
FPKM: Fragments Per Kilo bases per Million
FDR: Calculated the false discovery rate
FC: Fold change
RT-qPCR: Quantitative real-time PCR
ΔΔCt: Comparative Ct
