# Supplementary figures and images for: Small Auxin Up RNAs influence the distribution of indole-3-acetic acid and play a potential role in increasing seed size in Euryale ferox Salisb
Source: BMC Plant Biol. 2020 Jul 3;20:311. doi: 10.1186/s12870-020-02504-2 (PMC7333270; doi:10.1186/s12870-020-02504-2)

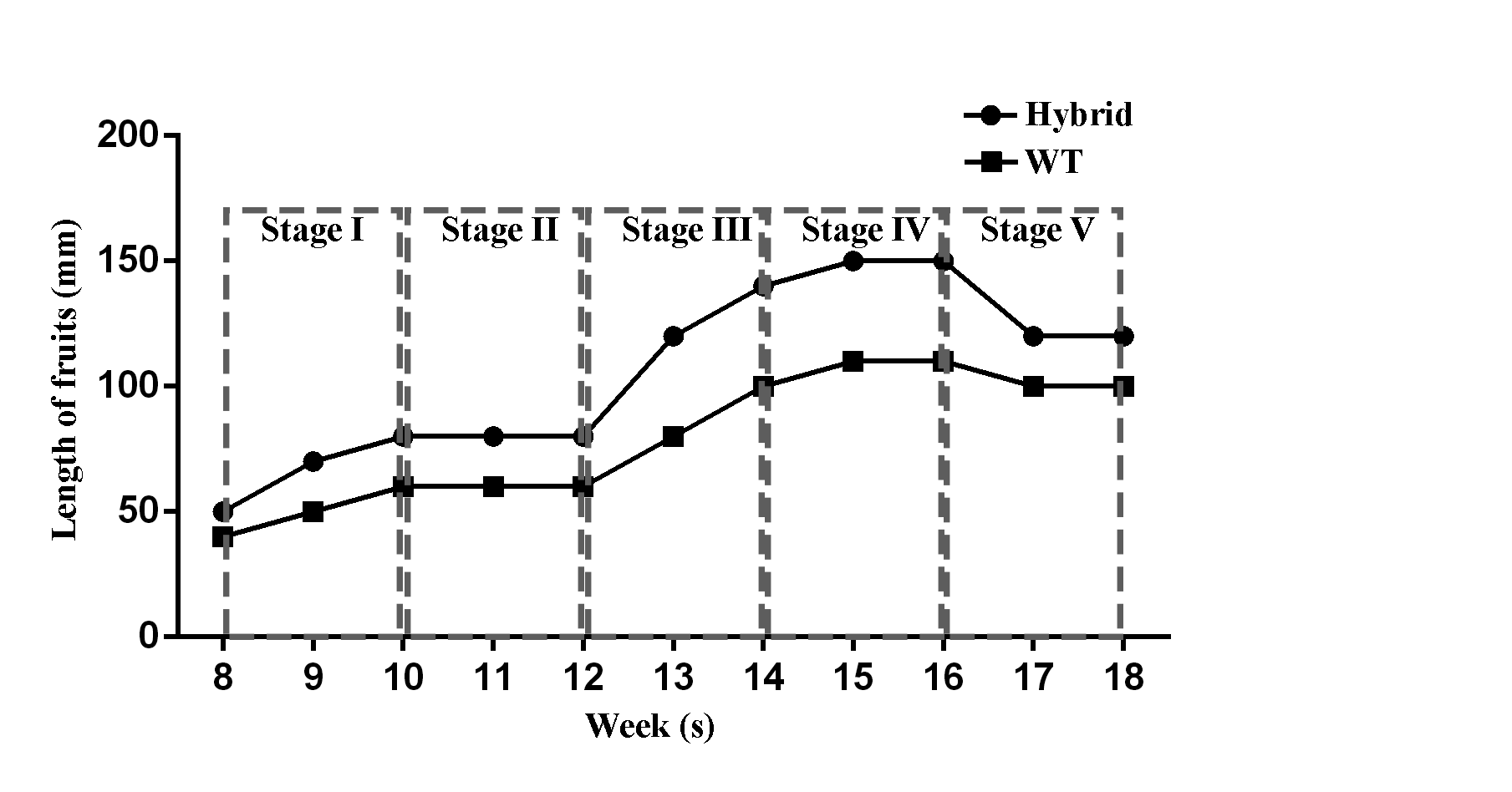

Supplement: Supplementary file 1 — Additional file 1: Figure S1. The development stage division of fruits. [file 12870_2020_2504_MOESM1_ESM.tif]

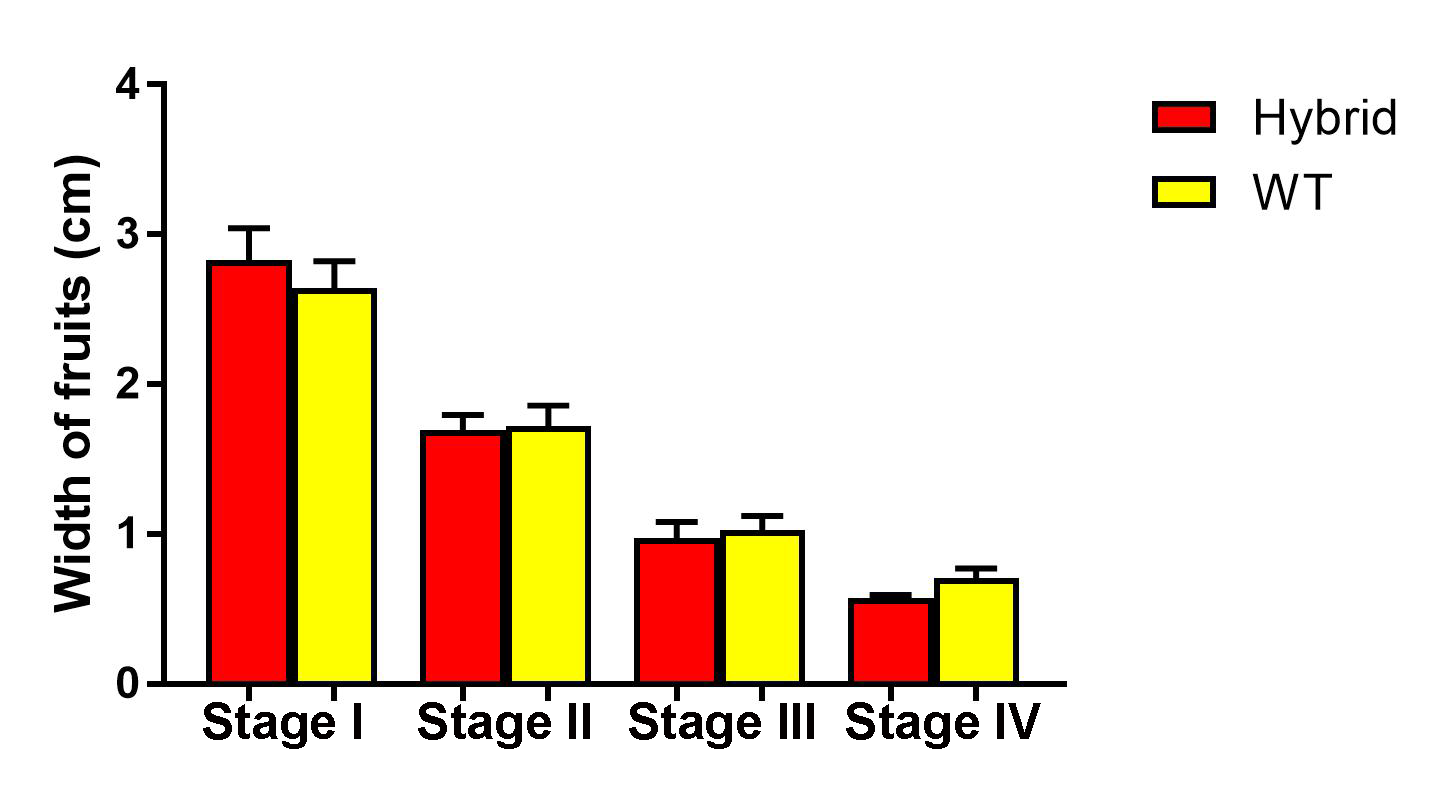

Supplement: Supplementary file 2 — Additional file 2: Figure S2. Width of fruits in HL and WT. [file 12870_2020_2504_MOESM2_ESM.tif]

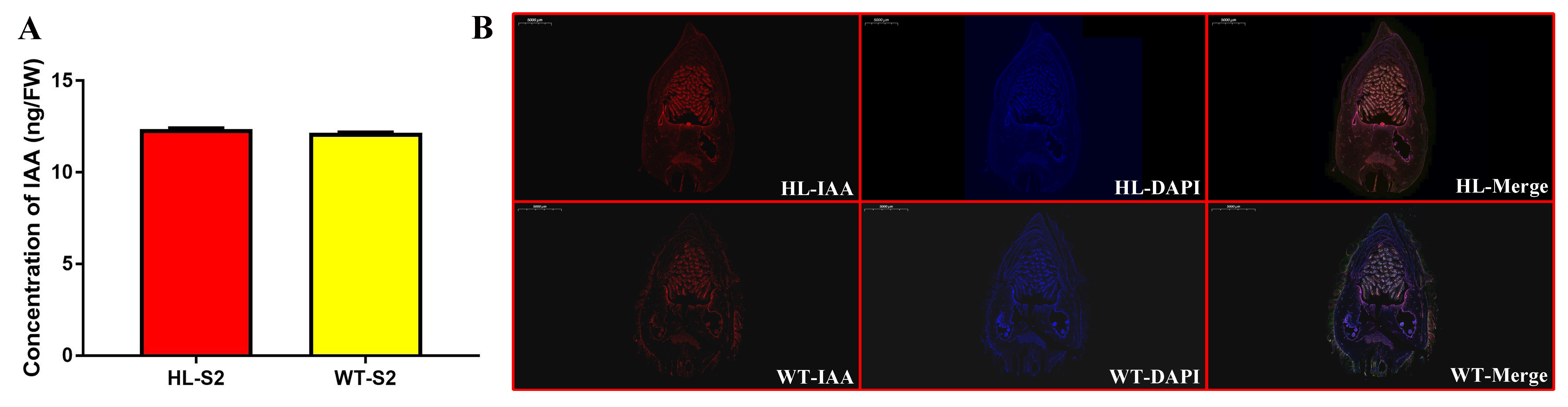

Supplement: Supplementary file 3 — Additional file 3: Figure S3. Concentration and distribution of IAA at stage II. [file 12870_2020_2504_MOESM3_ESM.jpg]

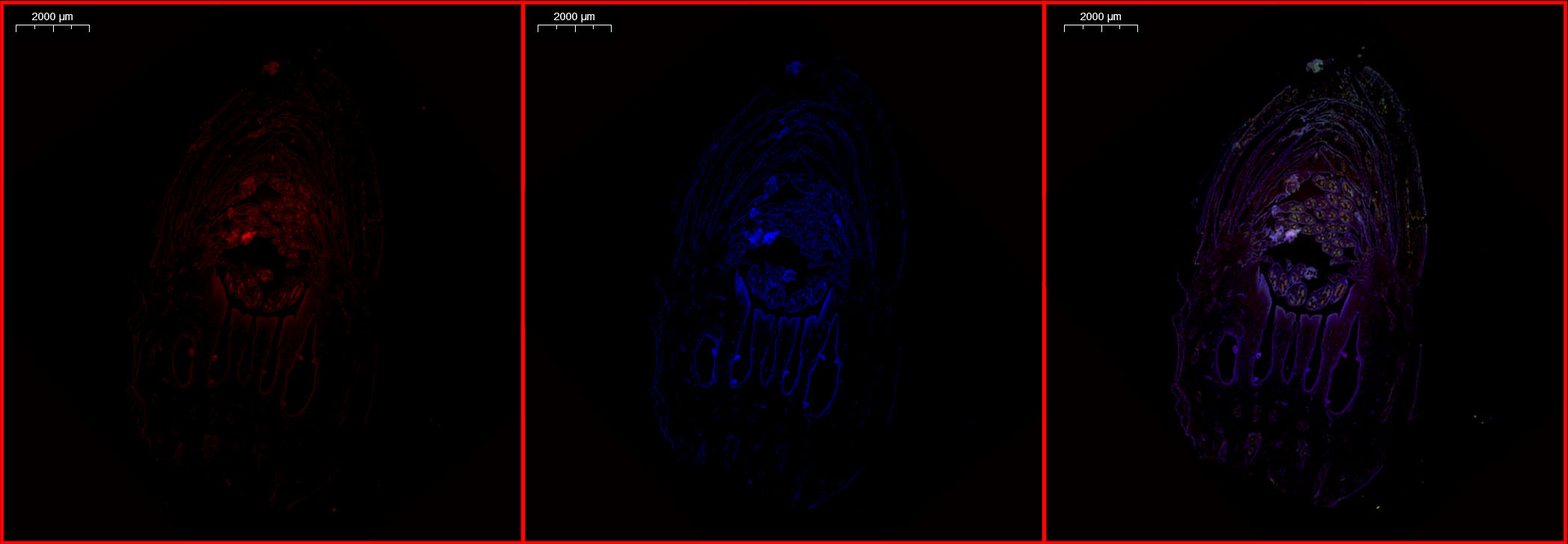

Supplement: Supplementary file 4 — Additional file 4: Figure S4. Distribution of IAA in fruits of SE. [file 12870_2020_2504_MOESM4_ESM.jpg]
